# Awake Percutaneous Cervical Cordotomy in Patients With Cancer: A Technical Report

**DOI:** 10.1111/papr.70159

**Published:** 2026-05-04

**Authors:** Adinda P. Pradhana, Hans Timmerman, Egbert J. M. Klarenbeek, Martijn L. Verstraaten, Ruud Stellema, Anna K. L. Reyners, André P. Wolff

**Affiliations:** ^1^ Department of Anesthesiology, Pain Center, University Medical Center Groningen University of Groningen Groningen the Netherlands; ^2^ Department of Medical Oncology, University Medical Center Groningen University of Groningen Groningen the Netherlands

**Keywords:** cordotomy, neoplasms, neurosurgical procedures, pain management, palliative care, spinothalamic tract

## Abstract

**Background:**

Percutaneous cervical cordotomy (PCC) is a minimally invasive radiofrequency procedure for intractable unilateral cancer pain. Commonly performed under conscious sedation, awake PCC enables real‐time patient feedback, potentially improving targeting precision and expanding eligibility for patients in whom sedation poses risks.

**Method and Technique:**

This article presents the structured protocol for awake PCC implemented at our center, including pre‐procedural training, positioning, fluoroscopic guidance, impedance monitoring, sensory and motoric testing, lesioning strategy, and aftercare. The key principle is to create the smallest effective lesion, applied incrementally until spinothalamic tract disruption is confirmed.

**Discussion:**

In our experience, awake PCC enables precise targeting and has yielded up to 90% success in pain relief. The approach requires substantial preparation, including training for both patient and team to ensure cooperation, clear communication, and tolerance of brief intraoperative discomfort. Although these demands can be challenging, we have found awake PCC to be a safe and effective last‐resort option for patients facing devastating pain.

**Conclusion:**

This technical note provides a practical and stepwise approach to awake PCC. It may assist clinicians in adopting or refining the technique.

## 1 Introduction

Percutaneous cervical cordotomy (PCC) is a minimally invasive procedure used to relieve pain by disrupting the lateral spinothalamic tract (STT) at the C1–C2 level of the cervical spinal cord [1, 2]. By creating lesions in this pathway, PCC interrupts pain and temperature transmission while preserving motor, touch, and gnostic functions, thereby providing effective pain relief [1, 2, 3, 4]. PCC is particularly indicated for patients with severe, unilateral, intractable pain located below the shoulder level due to cancer, and who have limited life expectancy (typically one to two years) [5]. While opioid therapy is commonly used for cancer pain management [6, 7], it may fail to provide adequate control in cases of refractory pain [6]. Moreover, side effects such as sedation, nausea, constipation, and cognitive impairment often compromise quality of life, especially in palliative settings [8, 9]. PCC offers sustained pain relief and helps reduce the need for opioid medications [2, 3, 10, 11].

PCC is commonly performed with conscious sedation [12, 13, 14]. However, it may compromise the clarity of intraoperative feedback, especially if the patient is not fully alert and cooperative, an issue that is particularly challenging in those receiving high‐dose opioid regimens [15]. There is also a risk of agitation or transient cognitive changes during the awake phase of this conscious sedation, which can interfere with sensory testing and place the lesion accurately [15, 16]. These risks are particularly relevant in older adults, frail patients, or those with significant comorbidities, where sedation may also increase the likelihood of respiratory depression, delirium, or delayed recovery [15, 17, 18]. In contrast, maintaining the patient's consciousness allows for more reliable sensory feedback and may improve targeting accuracy, especially when lesioning the somatotopically organized STT [2, 19]. It also expands access to patients for whom sedation poses clinical risks [20].

PCC is generally considered a safe procedure, though not entirely without risk [21]. Literature often cites neuropathic deafferentation pain as a potentially concerning long‐term complication, reported to occur rarely but with the possibility of persistence [3, 10, 22]. However, in over 250 cordotomy procedures performed at our center during the past decade, no confirmed cases have been encountered. Our annual caseload has increased from approximately five procedures in the early years to more than 50 in 2024. More commonly, mild and self‐limiting adverse effects were observed, including transient dysesthesia, localized intervention‐site pain, temporary motor weakness, and urinary retention.

This technical note outlines the approach at our center for performing awake PCC without conscious sedation, proposed as a potentially safer alternative. The goal is to provide a practical reference for clinicians seeking to implement or refine this technique.

### 1.1 Anatomical Considerations for PCC

The C1–C2 region is the only site for percutaneous cervical cordotomy due to its unique anatomy. The absence of an intervertebral disc and non‐overlapping laminae, with stability provided by the atlantoaxial ligaments, creates a wide interlaminar space that facilitates needle access and reduces the risk of procedural complications [23, 24]. Additionally, this level offers clear fluoroscopic reference points for needle guidance [23, 24, 25].

**FIGURE 1:**
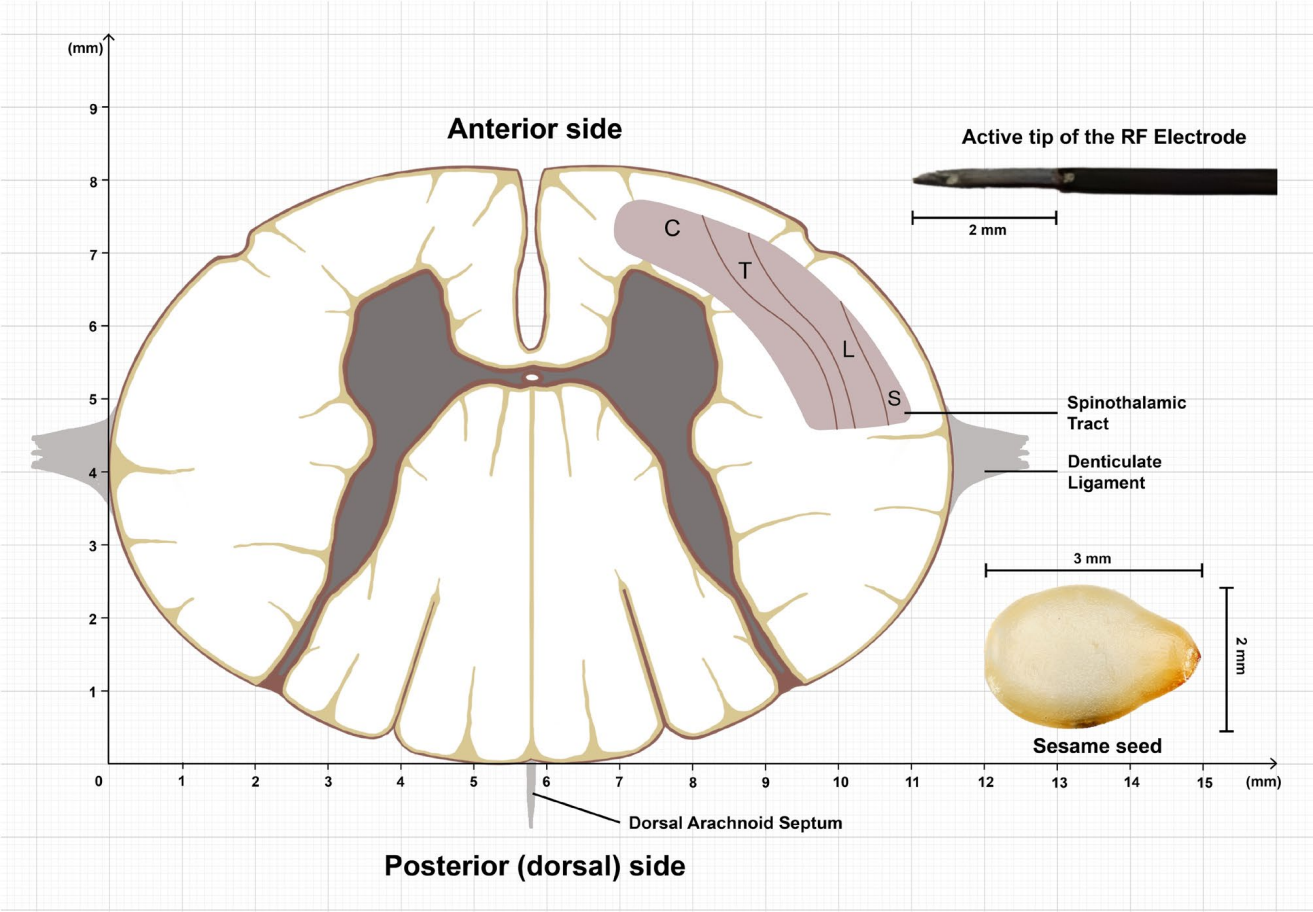
Scaled cross‐sectional illustration of the C2 cervical spinal cord highlighting the estimated area of the STT and its somatotopic organization, where fibers from the lower limb are located dorsolaterally, while those from the upper limb are positioned more anteromedially, which is represented proportionally to highlight relative differences between cervical, thoracic, lumbar, and sacral fibers. The distal active tip of the RF electrode (2 mm exposed) is included to illustrate its scale relative to the tract. The STT area (estimated 7.41–11.11 mm^2^) is shown in comparison to a single sesame seed (~4.71 mm^2^) to visualize scale. The millimeter grid provides spatial reference for lesion targeting during PCC. Illustration by A.P. Pradhana, created using Procreate (v5.3.15, Savage Interactive, Australia) and Adobe Illustrator 2025 (Adobe Inc., USA), based on established anatomical principles. Abbreviations: Mm, millimeters; C, cervical; T, thoracic; L, lumbar; S, sacral.

At the C1–C2 level, the spinal cord has an average cross‐sectional area of 74.06 mm^2^, measuring about 8.2 mm anteroposteriorly and 11.5 mm transversely on average [26]. STT occupies only 10%–15% (7.41–11.11 mm), highlighting the precision required for effective lesioning. At this level, the STT exhibits somatotopic organization, with lower limb fibers located dorsolaterally and upper limb fibers positioned anteromedially (Figure 1), allowing lesions to be tailored to the patient's pain distribution [2].

**FIGURE 2:**
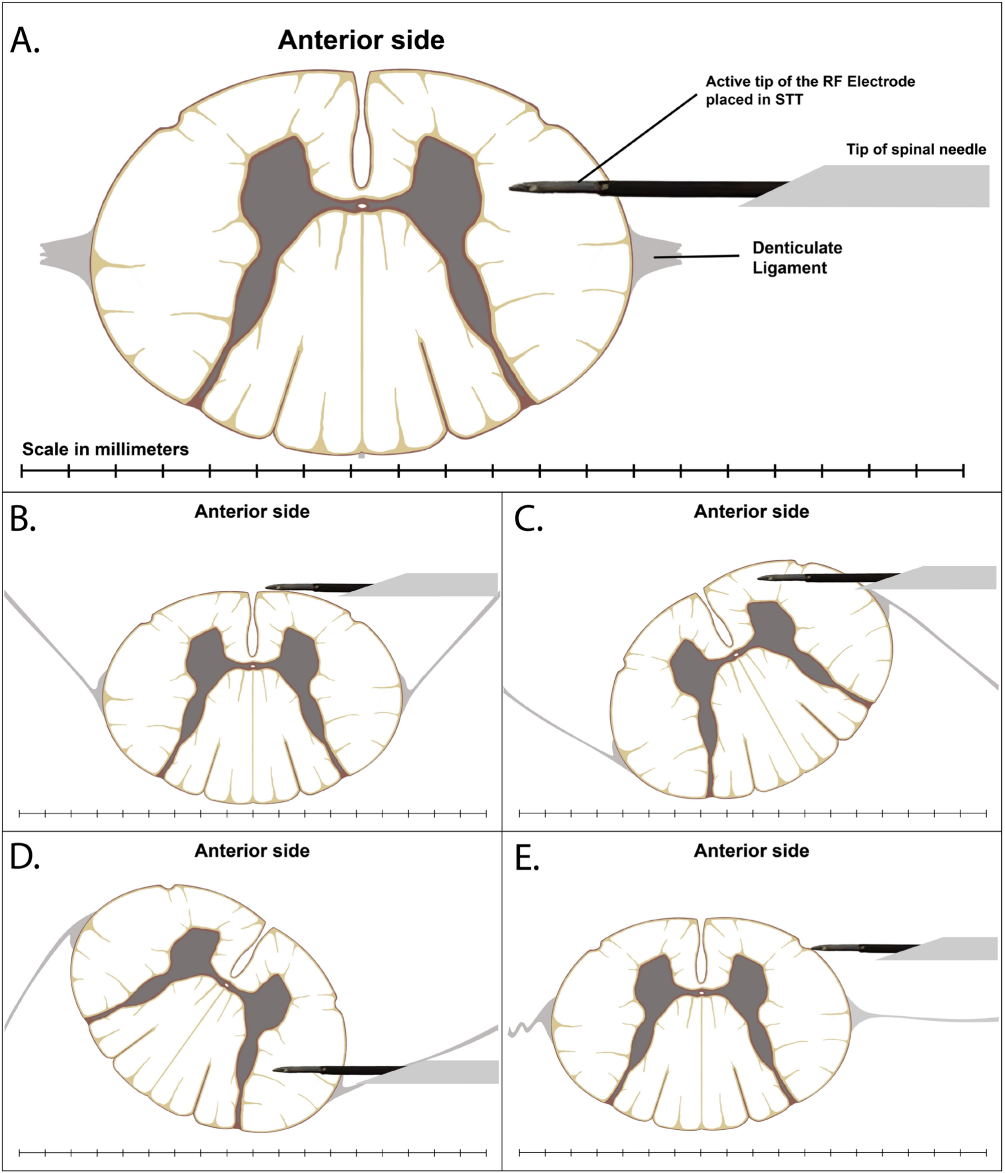
Illustration of the possible movements of the spinal cord within the subarachnoid space during percutaneous cervical cordotomy (PCC). (A) Cross‐section of the C2 cervical cord illustrating the ideal electrode placement: The distal active tip of the RF electrode passes smoothly through the pia. It lies within the STT under stable cord conditions. A millimeter scale reference is provided. (B–E) Examples of cord movement relative to the needle and electrode: Posterior movement (B), axial rotation with the lateral surface rotating anteriorly (C), axial rotation with the lateral surface rotating posteriorly (D), and lateral shift when the cord is pushed away by the needle (E). These variations open the possibilities that the electrode tip may lie more medially or even reach the contralateral dorsal cord. While the possible patterns of cord movement can be anticipated, the electrode position can only be approximated; definitive confirmation requires intraoperative sensory and motoric testing. The concept illustrated here was proposed by E.J.M. Klarenbeek, based on his experience and, in part, inspired by the findings of Strauss et al. [30] regarding spinal cord shift during PCC. The illustration was created by A.P. Pradhana using Procreate (v5.3.15, Savage Interactive, Australia) and Adobe Illustrator 2025 (Adobe Inc., USA), based on established anatomical principles.

At this level, the spinal cord is tethered laterally by the denticulate ligaments, which provide the primary stabilization [27, 28]. Additionally, delicate arachnoid septa (dorsal and dorsolateral) may offer minor additional support [29]. These structures limit mobility but do not abolish it, allowing cord shifts and partial rotations during dural and pial puncture that must be anticipated during PCC (Figure 2) [27, 28, 30].

For these reasons, PCC is primarily indicated for unilateral pain below the C4 dermatome. In higher cervical dermatomes (C1–C4), the corresponding STT fibers lie anteromedially [31], a region technically difficult to reach from a lateral approach because perpendicular entry through the pia mater is limited, and the cord tends to rotate under pressure [25, 28]. Additionally, pain above C5 is transmitted partly via the spinothalamic tract (C1–C4 dermatomes: posterior head and neck) and partly via the trigeminothalamic tract (fifth cranial nerve divisions V1–V3: face and anterior neck) [32, 33, 34]. Therefore, selective interruption of STT alone may be insufficient for pain above the C5 level. The detailed anatomical basis of the STT and related pathways is described in the Data S1 (Figure S1).

## 2 Method and Techniques

### 2.1 Patient Selection and Preoperative Preparations

#### 2.1.1 Indication

In our country, the Netherlands, awake PCC is considered within a clear palliative framework aimed at improving quality of life, including by reducing opioid use and its associated side effects. While the guidelines criteria are well established [1, 5], exceptions are possible when supported by clear clinical reasoning and documentation. At our center, PCC is considered particularly valuable in the palliative setting when the pain source is clearly nociceptive or peripheral neuropathic, as these mechanisms respond best to STT disruption.

#### 2.1.2 Contraindications

PCC is contraindicated in patients with coagulopathy, elevated intracranial pressure, bilateral pain, or a life expectancy of over two years [5]. Midline pain and visceral pain are relative contraindications due to potentially limited efficacy, as their response is less predictable compared to well‐localized unilateral somatic pain [1, 35]. Additionally, awake PCC may be unsuitable for patients unable to lie supine or those with cognitive impairment, severe anxiety, or other factors that limit cooperation during the procedure.

#### 2.1.3 Patient Preparations

The physician (typically an anesthesiologist or pain physician in our center) conducts a comprehensive evaluation to assess pain severity, review imaging for anatomical landmarks, and rule out contraindications. When necessary, medication adjustments (such as withholding anticoagulants) are made in consultation with the relevant specialist to minimize the risk of bleeding.

#### 2.1.4 Consent

Once eligibility is established, the procedure is discussed in detail. Patients must understand the results of irreversible sensory changes and weigh them against the expected pain relief. At our center, we present our results quantitatively to patients—approximately 90% success in pain relief, with major complications being rare—as they often find numbers more reassuring than general assurances. This transparency is central to building trust.

We emphasize that awake PCC is not comfortable. For safety, patients must remain awake, still, and may feel pain during lesioning, yet the lasting pain relief outweighs this discomfort. The procedure is continued only as long as necessary: until sufficient STT lesioning is achieved, the patient reaches their limit, or technical difficulties or complications require stopping. We never push beyond the patient's tolerance. Additionally, patients are informed that PCC is technically complex and may fail due to anatomical variation, difficulty targeting, or incomplete lesioning. If needed, it can be repeated after about a week, once edema and tissue changes have resolved.

To support decision‐making, patients are encouraged to take time and raise questions. Relatives may join discussions if doubts persist. In most cases, a decision is reached within a week, as candidates are typically in severe pain and view PCC as a last option. When the process takes longer, it is usually because the patient is not in urgent distress or ultimately chooses to decline. In such cases, the choice is respected and alternative options explored. However, if the patient consents, a training session is scheduled. The final decision on whether PCC can proceed depends on this training (i.e., the patient's ability to cooperate).

#### 2.1.5 Patient Procedural Training

**FIGURE 3:**
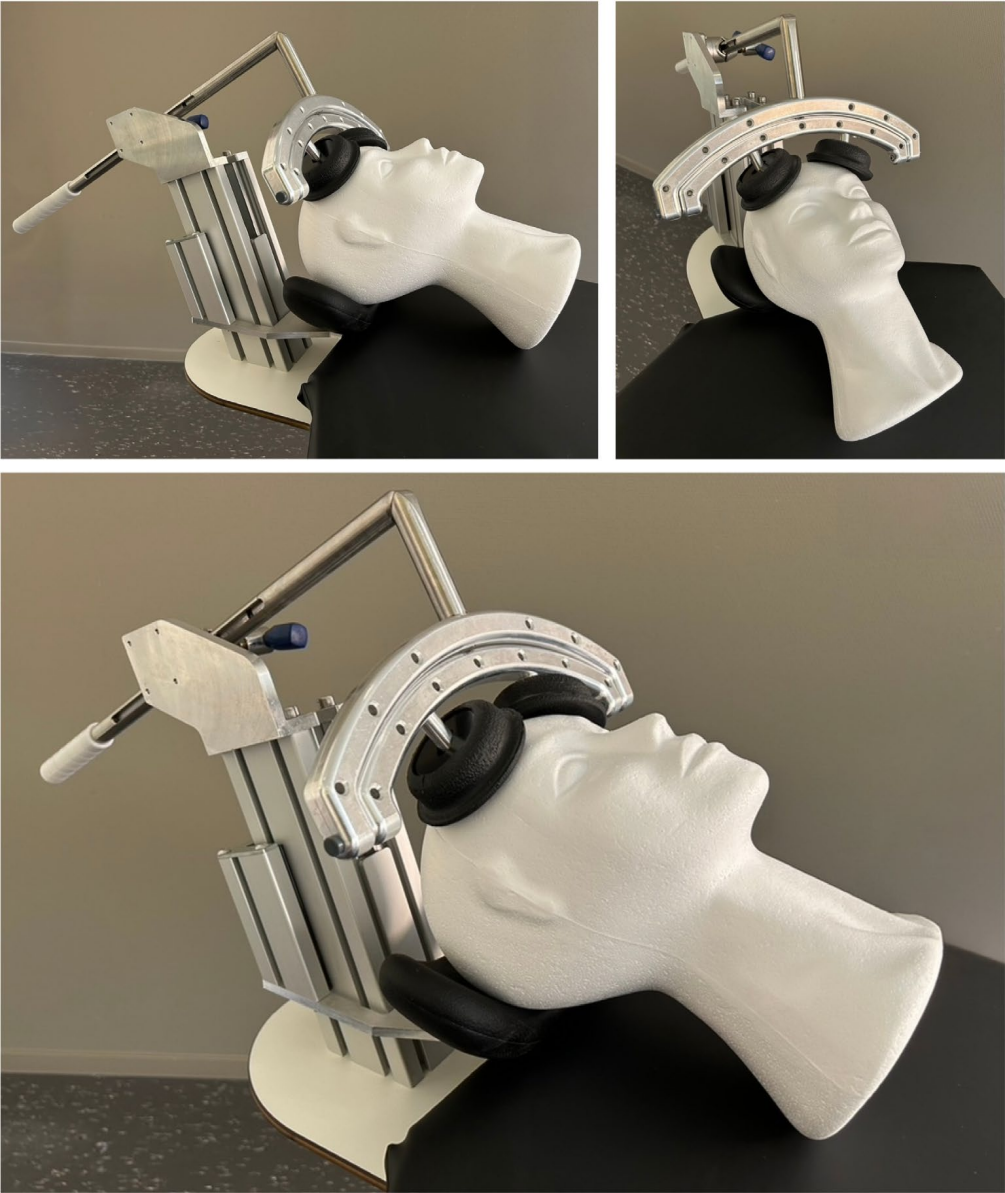
Demonstration of the Rosomoff head holder mounted on a mannequin head to illustrate positioning during percutaneous cervical cordotomy (PCC). Photograph taken by A.P. Pradhana and H. Timmerman in the cordotomy procedure room of the Pain Center, University Medical Center Groningen.

A training session is conducted one day before the procedure to familiarize both patient and care team. The patient visits the procedural room, meets the cordotomy team, and is introduced to key equipment. Positioning is practiced in the supine position with the Rosomoff head holder applied for stability (Figure 3). Baseline motor and coordination testing are performed and documented during this session. The patient is asked to lift and extend both arms against gravity, squeeze the physician's hands to assess grip strength, and raise each leg with knees bent while holding for 10 s. Coordination is tested by extending both arms laterally and touching the nose in a straight movement.

Patient feedback is the only way to confirm accurate electrode placement and adequacy of the lesion. The training, therefore, covers the entire procedure, including the ice‐cold and pin‐prick sensation tests, where patients learn to recognize sensations and communicate responses clearly. They are taught to provide concise, precise descriptions and avoid responding to motoric testing, which is objectively observable. This ensures efficient communication, as excessive talking may introduce unnecessary movement. Training also includes counting practice, where the team and patient count aloud each second to simulate lesion duration, allowing the patient to anticipate the duration to endure. This structured pre‐procedural preparation reduces anxiety, improves cooperation, and increases tolerance during the procedure [36].

The training is also important for the physician and the nurse responsible for patient care and sensory testing. Since sensory response behavior varies between individuals, this session builds trust, aligns expectations, and improves efficiency.

If, despite training, the patient cannot cooperate, does not understand the tasks, or shows inconsistent responses (i.e., due to central involvement), then PCC will not be performed, as this would increase risk and reduce effectiveness.

### 2.2 Technical Description

#### 2.2.1 Procedure Preparation

The awake PCC procedure is performed in a procedural room with continuous vital sign monitoring (including ECG, pulse oximetry, and non‐invasive blood pressure), oxygen, and emergency equipment readily available. The cordotomy team includes a physician who performs the procedure, two trained nurses (one dedicated to patient care and sensory testing, the other to instrument handling and documentation), and a radiographer responsible for operating the C‐arm fluoroscopy.

The Cosman G4 radiofrequency (RF) generator (Boston Scientific, USA) is used to perform impedance testing, nerve stimulation, and lesioning. A 20G spinal needle (Becton Dickinson, USA) is prepared for contrast injection to visualize anatomical landmarks. The dedicated RF electrode set (Boston Scientific, USA) consists of a 20G introducer spinal needle and a 28G RF electrode needle (11.7 cm), used to deliver the RF lesion. It is worth noting that electrode characteristics vary by manufacturer and may require different settings to achieve the desired results.

For local anesthesia, lidocaine HCl 1% (B. Braun, Germany) is prepared. We use Lipiodol Ultra Fluid (480 mg I/ml; Guerbet, France) as the contrast agent. Additionally, we arrange chlorhexidine gluconate 0.5% in 70% alcohol for skin antisepsis and a sterile procedural set which includes a sterile surgical gown, a sterile drape with a transparent central opening, a fluid container, a clamp, and an aseptic preparation set, along with ordinary disposable syringes (10 mL, 5 mL, and 3 mL), luer‐lock syringe plugs, and a three‐way stopcock with connection tubing. Lastly, a latex ice container pouch is prepared for cold sensation testing, accompanied by an ordinary sterile disposable syringe needle for pinprick testing to assess sensory response.

#### 2.2.2 Procedure Execution

A time‐out procedure is performed to verify the patient's identity, diagnosis, side (contralateral to the side of the pain), and procedural details, ensuring accuracy and safety. Once confirmed, the patient receives 2 g of cefazolin IV for prophylactic coverage against bacterial infection.

The patient is then positioned supine on the operating table. The head is kept in a neutral position with the Rosomoff head holder in place, and additional head support is available upon request to maintain neutrality while maximizing comfort. Continuous vital‐sign monitoring is applied, and baseline readings are recorded to ensure patient stability before proceeding. Supplemental oxygen is used only when clinically necessary.

#### 2.2.3 Fluoroscopy Setup and Needle Insertion

The physician stands at the patient's head, contralateral to the side of the pain, with the C‐arm positioned opposite. A lateral X‐ray visualizes the C1–C2 interspace, and the skin entry point is marked using sterile surgical ink. After aseptic preparation, 2–4 mL of lidocaine is injected subcutaneously, followed by 10 mL into deeper tissues near bony structures to ensure adequate anesthesia.

Afterward, a 20‐G spinal needle is inserted under fluoroscopic guidance, advancing toward the C1‐C2 interspace. The needle advances through the dura, where resistance is typically felt by the physician, which is often reported as a “pop” sensation upon puncture [37, 38, 39]. CSF outflow confirms dural penetration. If blood appears in the needle hub, repositioning is required before proceeding.

#### 2.2.4 Intrathecal Contrast Injection and Targeting the STT

After confirmation, a three‐way stopcock with an extension line is attached to the spinal needle. Subsequently, 3 mL of CSF, 3 mL of Lipiodol, and 3 mL of air are withdrawn into a 10 mL syringe and shaken to form a temporary suspension for even distribution. While the remaining mixture is kept for potential further use, a maximum of 2 mL is slowly injected intrathecally under real‐time fluoroscopy, just enough until three distinct lines appear in the image, representing:

1. The anterior border of the spinal cord.
2. The denticulate ligament.
3. The posterior border of the dura mater.

Real‐time fluoroscopy enables clear visualization of anatomical landmarks and ensures minimal use of Lipiodol, which cannot be cleared and may persist intrathecally for decades [40, 41].

**FIGURE 4:**
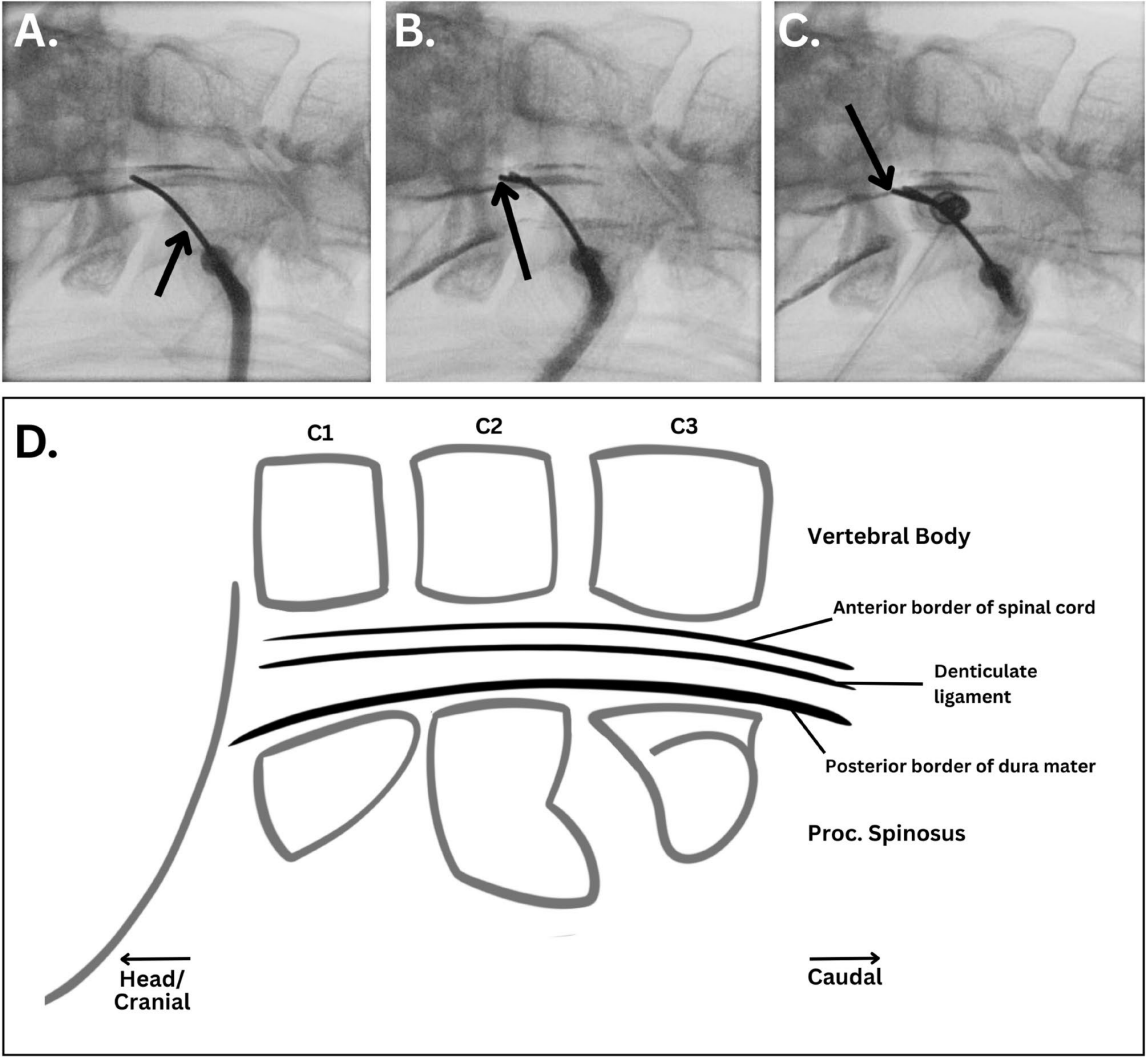
Fluoroscopic and schematic illustration of needle positioning during percutaneous cervical cordotomy (PCC). (A) The arrow indicates the initial spinal needle placement after insertion. Lipiodol is injected to visualize anatomical landmarks. (B) The contrast (lipiodol) spreads evenly, enhancing visualization of the subarachnoid space and dural margins. The arrow indicates the second spinal needle introduced based on the landmarks defined by the contrast. (C) The arrow indicates the final placement of the second spinal needle, positioned anterior to the denticulate ligament as intended. At this stage, the electrode is inserted, shown by the opaque hub at its head. (D) Schematic sagittal view of the cervical spine (C1–C3), illustrating key anatomical landmarks for needle guidance, including the posterior dura, denticulate ligament, and spinal cord border. The angle of the fluoroscopic projection causes the spinal needles to appear shorter and less distinct. Fluoroscopic image obtained during a cordotomy procedure performed by M.L Verstraaten. at the Pain Center, University Medical Center Groningen; patient data anonymized. Illustration created by A.P. Pradhana using Procreate (v5.3.15, Savage Interactive, Australia) and Canva (Canva Pty Ltd., Australia).

Once the denticulate ligament is visualized, the stopcock is closed, and a second spinal needle, the introductory needle, is inserted adjacent to the first one (Figure 4). Under C‐arm guidance, the needle is carefully advanced anterior to the denticulate ligament. After the second spinal needle penetrates the dura (confirmed by CSF outflow), an RF electrode is inserted through this introductory spinal needle to reach the target location based on somatotopic organization [12, 42].

#### 2.2.5 Impedance, Sensory, and Motoric Testing

Impedance monitoring guides electrode progression, ensuring accurate placement within the spinal cord. Typical impedance values include 200–300 Ω in CSF, around 400 Ω at the pia mater, and 800–1000 Ω within the spinal cord [2, 12, 42, 43]. Once impedance indicates that the electrode is within the cord, sensory stimulation is performed to confirm that the tip is in the STT. Because the target is the STT, sensory testing is performed first to confirm tract localization before motoric testing. Sensory testing is applied at 50 Hz with a 1 ms pulse width, gradually increasing the voltage in 0.01 V steps up to 0.2 V. In damaged cord (i.e., after lesioning), the threshold may be higher, and stimulation can be increased up to 0.4 V. Similarly, in conditions where sensibility is altered (e.g., patients receiving high‐dose opioids or gabapentinoids, or older patients), higher voltages may also be required. Contralateral temperature sensation within the patient's painful side is the most certain confirmation of precise electrode placement in the STT. If the expected response is absent, the needle is repositioned, and testing is repeated. In our experience, multiple intraparenchymal adjustments can usually be performed safely, whereas permanent injury arises primarily from lesioning.

Once sensory confirmation is achieved, motoric testing is performed to prevent lesioning of motor pathways. Low‐frequency stimulation (2 Hz, 1 ms pulse width) is applied, increasing in 0.1 V steps up to a maximum of 0.4 V. For the same reasons as in sensory testing, higher stimulation may be applied up to 0.6 V.

Ipsilateral trapezius contractions are common, likely due to stimulation affecting nearby cell bodies in the gray matter [19, 42]. The nurse responsible for patient care assists by holding the patient's thumb to detect subtle ipsilateral movements. The thenar muscle group is the most sensitive and specific indicator of corticospinal tract activation [19]. If repositioning is required, testing should be restarted from the beginning. Importantly, cord movement may result in only part of the active tip lying within the STT while the remainder extends into adjacent tracts (e.g., CST or contralateral STT). Understanding this concept is essential to connect patient feedback with the estimated position of the active tip (Figure 2).

### 2.3 Radiofrequency Ablation and Sensory Testing

Upon successful targeting, radiofrequency lesioning is typically performed at 90°C for 10 s, after a 10‐s ramp, using our electrode. Without repositioning the needle, sensory assessment is conducted using cold sensation testing and a sharp pinprick test to determine whether the targeted area of the STT has been effectively interrupted. Sensory testing is performed in a descending pattern, starting from the shoulder, chest, and upper extremities, and moving downward systematically, regardless of the dermatomal pattern. Sensory perception is always compared with the contralateral side. If necessary, additional lesioning can be performed either at the same position or with slight adjustments until one of the predefined stopping criteria is reached.

### 2.4 Aftercare and Monitoring

After the procedure, the insertion site is assessed for bleeding, and a sterile dressing is applied. Motor and coordination are retested to verify that no new immediate deficits have occurred.

The patient is monitored in recovery for at least 1 h, with attention to discomfort, unusual sensations, respiratory changes, and vital signs. Respiratory changes are of particular concern in patients previously receiving opioids, as reduced analgesic needs after lesioning may increase the risk of respiratory depression. Motor deficits from permanent tract injury present immediately, whereas those from temporary spinal cord edema appear later, helping distinguish permanent from transient complications.

Subsequently, the patient is admitted to the general ward for at least 24 h for continued observation and monitoring. Monitoring includes reassessment of motoric and sensory function, temperature perception, local pain at the intervention site, and bladder function, as temporary urinary retention may occur. A mild, transient temperature rise may also occur post‐procedure due to inflammation or disruption of thermoregulatory pathways [44, 45]. This typically appears immediately, whereas fever from infection usually develops later as part of the infectious process, and needs to be clearly distinguished.

## 3 Discussion

Awake PCC is a delicate procedure that demands technical precision, strong teamwork, and thorough preparation [21, 46]. We believe that success depends on a well‐coordinated team where each member understands their role and communicates effectively. It's essential not only to perform the procedure regularly but also to take the time to reflect, review the approach, and learn from each case. Team discussions, both formal and informal, help to identify areas for improvement. Additionally, patient feedback helps to improve the overall care. This continuous cycle of practice, reflection, and shared learning has been central to our effort to deliver awake PCC safely and effectively.

Awake PCC offers distinct advantages because the technique enhances targeting precision by enabling real‐time feedback during lesioning. A central principle in our practice is to create the smallest effective lesion that produces a measurable clinical effect of STT destruction. We therefore apply radiofrequency at 90°C for 10 s using our electrode, as we observe that lower temperatures tend to create lesions that are too small and difficult to monitor, while longer durations may result in a wider burn. By combining this with clear feedback from a fully conscious patient, we can obtain the intended effect while minimizing the risk of injuring adjacent areas. On average, we perform four lesions per patient, with a maximum of eight lesions sometimes required. Over the past decade, this stepwise method has helped us balance efficacy with safety. Furthermore, the absence of sedation simplifies management and decreases procedural risk, as sedation may compromise respiratory function. Maintaining spontaneous breathing is essential, since the procedure requires the patient to remain still in a supine position for about an hour and to communicate effectively throughout [1].

Despite its advantages, awake PCC also presents several challenges. The first is the considerable time required, most of which is devoted to preparation rather than the procedure itself. Patients must place profound trust in the team, often while facing fear and vulnerability, which demands a high level of compassionate communication and interpersonal competence. A second challenge is the pain patients may need to endure—not only from their underlying cancer but also during lesioning. Some experience distinct pain during the 10‐s burn, while others do not. However, this 10‐s pain is rarely the main concern, as patients already anticipate and accept this hardship, recognizing that it is worth the prospect of lasting relief in their remaining lifetime. More often, discomfort arises from their original cancer pain, which may worsen with positioning. We do everything possible to ease this without compromising consciousness, occasionally administering a small opioid dose if needed.

Sedation may be useful in selected cases, for instance, in patients unable to tolerate lying supine or with severe anxiety [47]. Yet our practice has shown that maintaining full consciousness provides the most reliable confirmation of correct electrode placement in the STT, as even light sedation can obscure feedback and increase the risk of imprecise targeting. This is particularly important, as the intended lesion must be both minimal and highly precise. Whether sedated PCC can achieve comparable accuracy requires further study.

Based on our experience, transient ipsilateral motor weakness is the most common adverse effect, typically attributed to local edema, and is usually mild and self‐limiting [22]. Other postoperative effects include temporary bladder dysfunction, slight postural imbalance, and Horner syndrome, which is likely due to incidental disruption of sympathetic fibers at the high cervical level [48], are generally benign. Respiratory compromise is often cited as a concern. But this should not discourage the use of PCC, as the procedure—particularly when performed unilaterally—does not impair respiratory function [49]. Unmasking of pre‐existing symptoms such as fatigue, dyspnea, or dysesthesia is also frequently encountered. Mirror pain—a rare but recognized complication—has been observed in approximately 3%–5% of our cases and is typically transient. Its pathophysiology is not fully clear but may involve the interplay of other tracts, bilateral innervation, and inhibitory pathways [50, 51]. Additionally, we have observed irreversible paresis, which occurred in less than 1% of cases.

## 4 Conclusion

Awake PCC represents a technically demanding but highly effective intervention for unilateral cancer pain in the palliative phase. By combining incremental lesioning with real‐time patient feedback, the procedure enables precise targeting of the spinothalamic tract while minimizing collateral injury. Although it requires significant preparation, patient cooperation, and careful intraoperative communication, the approach necessitates no sedation, thereby potentially expanding eligibility to most patients. When integrated into a multidisciplinary palliative care strategy, awake PCC offers patients profound relief at the end of life, justifying the effort it demands.

## Author Contributions

A.P.P. initiated, outlined, and led the development of the article, coordinated input from all authors, integrated feedback, and prepared the illustrations. H.T. supervised the work, provided continuous guidance on the drafting process, and critically reviewed the text in detail to ensure clarity and precision. E.J.M.K. contributed to the conceptual development of the technical approach, provided procedural consultation and perspective, and critically reviewed the manuscript. M.L.V. contributed to the conceptual and procedural aspects, provided technical guidance during the early development of the article, and reviewed the manuscript. R.S. contributed to the procedure, offered technical input, and reviewed the manuscript. A.K.L.R. supervised the work, provided guidance on oncological and palliative care perspectives, and critically reviewed the manuscript. A.P.W. supervised the work, provided guidance on clinical and procedural aspects, and critically reviewed the manuscript. All authors approved the final version.

## Funding

This work received no specific grant from any funding agency in the public, commercial, or not‐for‐profit sectors.

## Ethics Statement

The authors have nothing to report.

## Consent

Written informed consent was not required, as this article is fully anonymized and contains no identifiable patient information, in accordance with institutional and journal ethical standards.

## Conflicts of Interest

The authors declare no conflicts of interest.

## Supporting information


**Figure S1:** Schematic representation of the Ascending spinothalamic pain pathway with major projections to cortical, subcortical, and brainstem regions involved in sensory, cognitive, and emotional processing of pain. Illustration by A.P. Pradhana, created using Procreate (v5.3.15, Savage Interactive, Australia) and Adobe Illustrator 2025 (Adobe Inc., USA), based on established anatomical principles. S1, primary somatosensory cortex; S2, secondary somatosensory cortex; PB, parabrachial nucleus; PAG, periaqueductal gray.

**Data S1:** Central pain pathways relevant to percutaneous cervical cordotomy.

## Data Availability

Data sharing is not applicable to this article as no datasets were generated or analyzed during the current study.
