# Supplementary figures and images for: Awake Percutaneous Cervical Cordotomy in Patients With Cancer: A Technical Report
Source: Pain Pract. 2026 May 4;26:e70159. doi: 10.1111/papr.70159 (PMC13138373; doi:10.1111/papr.70159)

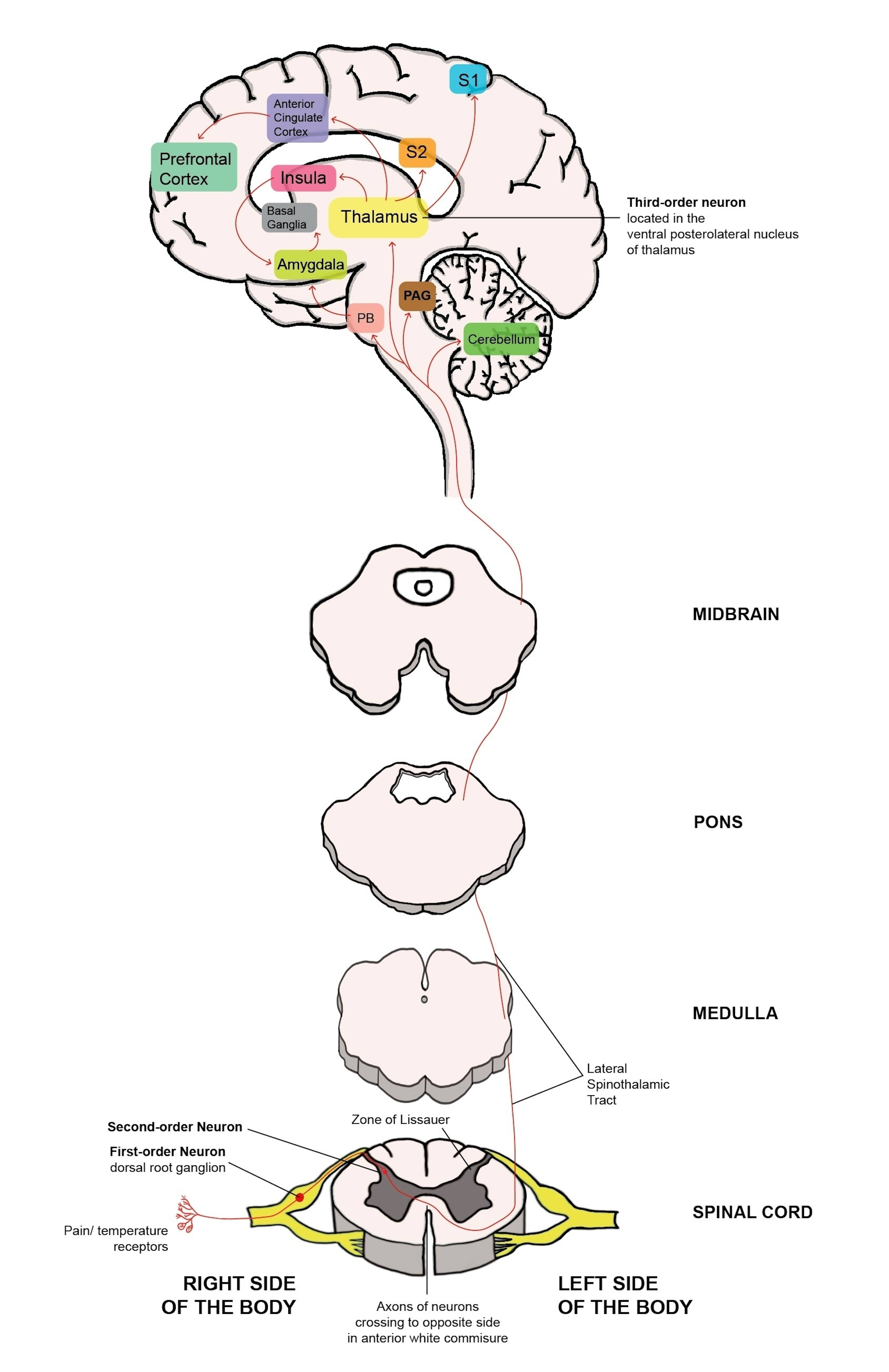

Supplement: Supplementary file 1 — Figure S1: Schematic representation of the Ascending spinothalamic pain pathway with major projections to cortical, subcortical, and brainstem regions involved in sensory, cognitive, and emotional processing of pain. Illustration by A.P. Pradhana, created using Procreate (v5.3.15, Savage Interactive, Australia) and Adobe Illustrator 2025 (Adobe Inc., USA), based on established anatomical principles. S1, primary somatosensory cortex; S2, secondary somatosensory cortex; PB, parabrachial nucleus; PAG, periaqueductal gray. [file PAPR-26-0-s002.tiff]
