# Supplementary material for: Awake Percutaneous Cervical Cordotomy in Patients With Cancer: A Technical Report
Source: Pain Pract. 2026 May 4;26:e70159. doi: 10.1111/papr.70159 (PMC13138373; doi:10.1111/papr.70159)
Supplement: Supplementary file 2 — Data S1: Central pain pathways relevant to percutaneous cervical cordotomy. [file PAPR-26-0-s001.pdf]

SUPPLEMENTARY MATERIAL S1

Central Pain Pathways Relevant to Percutaneous Cervical Cordotomy

The term nociception refers to the neural processes by which the sensory nervous system detects and encodes noxious stimuli that signal actual or potential tissue damage.[1,2] Noxious stimuli are environmental or internal signals that threaten tissue integrity and are capable of activating nociceptors. They may be mechanical (resulting from excessive pressure or tissue distortion), thermal (caused by extreme heat or cold), or chemical (due to irritants or inflammatory mediators such as protons, bradykinin, or prostaglandins).[3,4] Nociceptive signaling originates in specialized free nerve endings distributed throughout the skin, muscles, joints, periosteum, and viscera.[5] These receptors transmit signals through thinly myelinated Aδ fibers, which mediate sharp, well-localized pain, and unmyelinated C fibers, which mediate slow, diffuse, burning pain. The cell bodies of these first-order neurons reside in the dorsal root ganglia, and their central axons enter the spinal cord via the dorsal roots to synapse with second-order neurons within Rexed laminae I, II, and V of the dorsal horn.[6–8] Before synapsing in the dorsal horn, many fibers ascend or descend a short distance in Lissauer’s tract, allowing nociceptive input to spread across adjacent segments above and below the level of entry.[6]

In contrast, nociceptive input from the face and anterior neck follows the trigeminal system.[9] First-order neurons are located in the trigeminal (Gasserian) ganglion, and their central axons enter the brainstem at the level of the pons. They descend in the spinal trigeminal tract and terminate in the spinal trigeminal nucleus, where they synapse with second-order neurons. These second-order neurons decussate and ascend in the contralateral trigeminothalamic tract, projecting to the ventral posteromedial (VPM) nucleus of the thalamus, which in turn relays signals to the primary and secondary somatosensory cortices and limbic regions.[10–12] However,

because this pathway conveys pain above C5, which lies outside the indication for percutaneous cervical cordotomy (PCC), it will not be discussed further here.

Within the dorsal horn, second-order neurons of the spinothalamic tract (STT) decussate via the ventral white commissure and ascend contralaterally in the anterolateral funiculus. At the C1–C2 level, the target region for PCC, the STT shows a somatotopic arrangement with sacral and lumbar fibers positioned dorsolaterally and cervical fibers more anteromedially.[13] This organization enables lesioning to be tailored to the patient's pain distribution, which is critical for PCC. Although the general somatotopy is well recognized, it is described with many variations, with some reports suggesting that pain and thermal fibers may be positioned separately within the tract.[14,15]

These fibers project to third-order neurons in the ventral posterolateral (VPL) nucleus of the thalamus, which then relay nociceptive input to multiple cortical and subcortical regions. The primary somatosensory cortex (S1) encodes localization and intensity, while the secondary somatosensory cortex (S2) contributes to bilateral integration and higher-order sensory processing.[5,16–18] The insula integrates pain with interoceptive awareness, shaping the subjective experience of discomfort, and via its connections to the amygdala and basal ganglia, further links pain to emotion (i.e., fear and anxiety) and motor readiness.[19,20] The anterior cingulate cortex (ACC) mediates the affective–motivational dimension, directing attention and behavioral responses to pain, and also projects to the prefrontal cortex (PFC) to influence decision-making and executive control.[19,20]

Additional projections highlight the integrative nature of pain processing. The cerebellum receives nociceptive input and contributes to sensorimotor coordination and the anticipation of painful stimuli.[21] The parabrachial nucleus (PB) relays signals not only to the thalamus but also directly to the amygdala, reinforcing the emotional salience of pain.[19,22,23] The periaqueductal gray (PAG) participates in descending pain modulation, while also receiving ascending nociceptive input, thereby integrating

sensory and affective dimensions to initiate defensive behaviors. Together, these widespread projections explain why pain is not only a sensory event but also an interoceptive, cognitive, and emotional experience. This organization is presented schematically in Figure S1.

[FIGURE S1]

References

1. Middleton SJ, Barry AM, Comini M, et al. Studying human nociceptors: from fundamentals to clinic. *Brain*. 2021;144(5):1312-1335. doi:10.1093/BRAIN/AWAB048
2. Armstrong SA, Herr MJ. Physiology, Nociception. *StatPearls*. Published online May 1, 2023. Accessed September 16, 2025. https://www.ncbi.nlm.nih.gov/books/NBK551562/
3. García-Domínguez M. Injury-Driven Structural and Molecular Modifications in Nociceptors. *Biology 2025, Vol 14, Page 788*. 2025;14(7):788. doi:10.3390/BIOLOGY14070788
4. Pinho-Ribeiro FA, Verri WA, Chiu IM. Nociceptor Sensory Neuron-Immune Interactions in Pain and Inflammation. *Trends Immunol*. 2016;38(1):5. doi:10.1016/J.IT.2016.10.001
5. Bourne S, Machado AG, Nagel SJ. Basic anatomy and physiology of pain pathways. *Neurosurg Clin N Am*. 2014;25(4):629-638. doi:10.1016/J.NEC.2014.06.001
6. Purves D, Augustine GJ, Fitzpatrick D, et al. Central Pain Pathways: The Spinothalamic Tract. Published online 2001. Accessed September 16, 2025. https://www.ncbi.nlm.nih.gov/books/NBK10967/
7. Duan B, Cheng L, Ma Q. Spinal Circuits Transmitting Mechanical Pain and Itch. *Neurosci Bull*. 2018;34(1):186-193. doi:10.1007/S12264-017-0136-Z/FIGURES/4
8. Karcz M, Abd-Elsayed A, Chakravarthy K, et al. Pathophysiology of Pain and Mechanisms of Neuromodulation: A Narrative Review (A Neuron Project). *J Pain Res*. 2024;17:3757. doi:10.2147/JPR.S475351
9. Kim HK;, Chung KM;, Xing J;, et al. The Trigeminal Sensory System and Orofacial Pain. *International Journal of Molecular Sciences 2024, Vol 25, Page 11306*. 2024;25(20):11306. doi:10.3390/IJMS252011306
10. Patel NM, Jozsa F, Das JM. Neuroanatomy, Spinal Trigeminal Nucleus. *StatPearls*. Published online September 10, 2024. Accessed September 15, 2025. https://www.ncbi.nlm.nih.gov/books/NBK539729/
11. Andrew DLE, May PJ, Warren S. Morphologic Characterization of Trigeminothalamic Terminal Arbors Arising From the Principal Nucleus in the Macaque. *Front Neuroanat*. 2020;14:562673. doi:10.3389/FNANA.2020.562673
12. Henssen DJHA, Pritsch C, Nazari P, Mulleners W, Vissers K. The non-decussating and decussating trigeminothalamic tracts in humans: A

combination of connectome-based tractography and histological validation. *Cephalalgia*. 2024;44(4). doi:10.1177/03331024241235168
13. Michael Honey C, Ivanishvili Z, Honey CR, Heran MKS. Somatotopic organization of the human spinothalamic tract: in vivo computed tomography–guided mapping in awake patients undergoing cordotomy. *J Neurosurg Spine*. 2019;30(5):722-728. doi:10.3171/2018.11.SPINE18172
14. Sharma ML, Marley K, McGlone FP, Gupta M, Marshall AG. Dissociation of Spinothalamic Modalities Following Anterolateral Cordotomy. *Canadian Journal of Neurological Sciences*. 2018;45(3):354-356. doi:10.1017/CJN.2017.290
15. Vedantam A, Bruera E, Hess KR, Dougherty PM, Viswanathan A. Somatotopy and Organization of Spinothalamic Tracts in the Human Cervical Spinal Cord. *Clin Neurosurg*. 2019;84(6):E311-E317. doi:10.1093/NEUROS/NYY330
16. Taren JA, Davis R, Crosby EC. Target physiologic corroboration in stereotaxic cervical cordotomy. *J Neurosurg*. 1969;30(5):569-584. doi:10.3171/JNS.1969.30.5.0569
17. Willis WD, Westlund KN. Neuroanatomy of the Pain System and of the Pathways That Modulate Pain. *J Clin Neurophysiol*. 1997;14(1):2. doi:10.1097/00004691-199701000-00002
18. Neugebauer V. Amygdala physiology in pain. *Handb Behav Neurosci*. 2020;26:101. doi:10.1016/B978-0-12-815134-1.00004-0
19. Baccei ML, Fitsgerald M. Development of Pain Pathways and Mechanisms. In: McMahon SB, Koltzenburg M, Tracey I, Turk D, eds. *Wall & Melzack's Textbook of Pain*. 6th ed. Saunders, Elsevier; 2013:143-155.
20. Craig AD. How do you feel? Interoception: The sense of the physiological condition of the body. *Nat Rev Neurosci*. 2002;3(8):655-666. doi:10.1038/NRN894;KWRD

SUPPLEMENTARY FIGURE LEGENDS

Figure S1. *Schematic representation of the Ascending spinothalamic pain pathway with major projections to cortical, subcortical, and brainstem regions involved in sensory, cognitive, and emotional processing of pain. Illustration by A.P. Pradhana, created using Procreate (v5.3.15, Savage Interactive, Australia) and Adobe Illustrator 2025 (Adobe Inc., USA), based on established anatomical principles. Abbreviations: S1 = primary somatosensory cortex; S2 = secondary somatosensory cortex; PB = parabrachial nucleus; PAG = periaqueductal gray.*
